# A systematic review of the effectiveness of policies restricting access to pregabalin

**DOI:** 10.1186/s12913-017-2503-x

**Published:** 2017-08-25

**Authors:** Brett R. Stacey, Jonathan Liss, Regina Behar, Alesia Sadosky, Bruce Parsons, Elizabeth T. Masters, Patrick Hlavacek

**Affiliations:** 1 Center for Pain Relief at UWMC-Roosevelt, University of Washington, 4225 Roosevelt Way NE, Seattle, WA 98105 USA; 2 Medical Research and Health Education Foundation, 7196 North Lake Drive, Suite A, Columbus, GA 31909 USA; 3 Pfizer Inc, 235 East 42nd Street, New York, NY 10017 USA

**Keywords:** Step therapy, Restriction policies, Prior authorization, Pregabalin

## Abstract

**Background:**

Formularies often employ restriction policies to reduce pharmacy costs. Pregabalin, an alpha-2-delta ligand, is approved for treatment of fibromyalgia (FM); neuropathic pain (NeP) due to postherpetic neuralgia (PHN), diabetic peripheral neuropathy (pDPN), spinal cord injury; and as adjunct therapy for partial onset seizures. Pregabalin is endorsed as first-line therapy for these indications by several US and EU medical professional societies. However, restriction policies such as prior authorization (PA) and step therapy (ST) often favor less costly generic pain medications over pregabalin.

**Methods:**

A structured literature search (PubMed, past 11 years) was conducted to evaluate whether restriction policies against pregabalin support real-world economic and healthcare utilization benefits.

**Results:**

Search criteria identified three claims analyses and a modeling study that evaluated patients with NeP and/or FM with and without PA restrictions; three other studies included patients with FM and NeP in plans with ST requirements, and one evaluated a mail order requirement program. All studies evaluated outcomes during follow-up periods of 6 months or longer. Overall, PA, ST, and mail order restriction policies effectively reduced pregabalin usage, but the effects were inconsistent with reducing pharmacy costs and were non-significant for total disease-related medical costs. Two studies (one PA; one ST) reported significantly higher disease-related costs in restricted plans. The modeling study failed to demonstrate cost savings with PA. Opioid usage was higher in PA-restricted plans (two studies). The US Centers for Disease Control and Prevention and several professional NeP guidelines recommend opioid use only in cases when other non-opioid pain therapies have proven ineffective. New US Government taskforce guidelines now seek to reduce opioid exposure. Additionally, in both ST studies, gabapentin utilization (a common ST edit) was significantly increased. Both studies had substantial proportions of FM and pDPN patients and the only pain condition gabapentin is approved to treat in the United States is PHN.

**Conclusion:**

PA and ST restriction policies significantly decrease utilization of pregabalin, but do not consistently demonstrate cost savings for US health plans. More research is needed to evaluate whether these policies may lead to increased opioid usage as found in some studies.

**Trial registration:**

N/A.

**Electronic supplementary material:**

The online version of this article (doi:10.1186/s12913-017-2503-x) contains supplementary material, which is available to authorized users.

## Background

In 2011, the Institute of Medicine (IOM) of the National Academies reported that approximately 100 million Americans suffered from chronic pain, which totals more than those who suffer from heart disease, cancer, and diabetes combined [1]. The IOM also estimated the associated national economic impact of chronic pain-related medical costs and lost productivity to be $635 billion annually. One study estimated per patient annual costs of neuropathic pain (NeP) in the United States to be $6016 (direct costs to payers), $2219 (direct costs to patients), and $19,000 (indirect costs including lost productivity) [2]. Epilepsy is another disorder with high prevalence and associated costs in the United States. In 2013, 4.3 million adults and 0.75 million children in the United States were diagnosed with epilepsy or a seizure disorder, with estimated direct and indirect costs associated with epilepsy of $15.5 billion [3].

The high costs and impact on patients’ health and quality of life underscore the need for efficacious and appropriate treatment of chronic pain and epilepsy. Pregabalin is a pharmacotherapy that is approved in the United States for treatment of NeP associated with post-herpetic neuralgia (PHN), painful diabetic peripheral neuropathy (pDPN), and spinal cord injury, as well as for fibromyalgia (FM) and as adjunct therapy for partial onset seizures [4]. Pregabalin is a calcium channel alpha-2-delta ligand drug and has been recommended as a first-line therapy by the International Association for the Study of Pain (IASP) treatment guidelines for NeP [5]. These guidelines are endorsed by multiple pain specialty organizations including the American Pain Society [6, 7]. Pregabalin has also been recommended as first-line therapy for the treatment of pDPN by the American Academy of Neurology, the American Association of Neuromuscular and Electrodiagnostic Medicine, and the American Academy of Physical Medicine and Rehabilitation [8].

Published studies support that treatment with pregabalin can be cost saving for FM. In a non-comparative model of clinical trial data, the pregabalin doses indicated for FM in the US prescribing information (300 and 450 mg/d) [4] resulted in overall lower mean annualized total costs than placebo [9]. In addition, a meta-analysis of pregabalin clinical trial data found that pregabalin treatment (vs. placebo) in patients with FM resulted in significantly fewer days of work lost [10]. In regard to partial onset seizures, using a 1-year hypothetical model, pregabalin as an adjunct therapy resulted in 23.8 additional seizure-free days per year with an incremental mean cost per day gained of $28.45 (cost per quality-adjusted life year $52,893), which is comparable with other anti-epileptic drugs [11].

In spite of evidence of efficacy, safety, and potential cost savings of pregabalin, many payers use formulary restriction policies such as prior authorization (PA), step therapy (ST), mail order requirements, and member cost sharing as a strategy to limit patient access to pregabalin. PA requires a healthcare provider or patient to submit a request for drug reimbursement to check if certain predetermined requirements are met before the payer will cover the drug. This can restrict access to a medication. PA policies have been reported to reduce expenditures on and utilization of the restricted drugs, but the impact on total medical costs and quality of patient care is less clear [12–16]. ST policies reimburse a medication only if a patient has tried and failed another therapy first. These ST edits are determined by the payer, and usually require the use of generic medications to lower pharmacy costs. In the case of pregabalin, many payers require ST edits through generic gabapentin or duloxetine. Gabapentin is indicated for PHN and as adjunct therapy for partial onset seizures [17–19], and duloxetine is indicated for pDPN, FM, and chronic musculoskeletal pain [20]. Mail order requirements restrict access of reimbursed drugs to postal delivery, and some of these mail order programs allow patients to use a generic alternative medication if they prefer to pick up their medications at a local pharmacy with a physical location.

Implementation of such restriction policies may be driven by the increasing costs of prescription medications in general. Indeed, prescription drug spending in the United States was higher ($858 per capita) than that of 19 other industrialized nations (average $400) [21]. The Assistant Secretary for Planning and Evaluation (ASPE) of the US Department of Health and Human Services reported that retail drug sales accounted for $328 billion out of $457 billion dollars of prescription drug spending in the United States in 2015 [22]. Overall, this total drug spending accounted for 16.7% of the costs of all personal healthcare services. The ASPE also reported a 15% rise in drug spending from 2010 through 2014, while economy-wide inflation rose 7%. They estimated that this rise in drug spending could be due to the following factors: 10% related to population growth, 30% because of increased number of prescriptions per person, 30% because of general economy-wide inflation, and 30% related to higher costs of drugs (either more prescribing of higher cost drugs or increases in prices). Like other drugs, the cost of pregabalin has increased too. However, drug costs alone are not enough to determine whether or not restriction policies should be implemented for a medication. The potential health benefits and related cost savings of a drug must also be considered.

In this literature review, we summarize published health economic studies on restriction policies for pregabalin and assess whether or not these restrictions resulted in real-world cost savings or health benefits.

## Methods

A systematic structured literature search of PubMed was conducted for publications related to pregabalin restriction polices in accordance with the PRISMA guideline for systematic reviews and meta-analyses [23]. Table 1 lists all search terms. Alternative forms of the search terms (e.g. plural, singular, different verb tenses) and alternative spellings were included in the search strategy (e.g. US vs. UK spelling, “healthcare” vs. “health care”). The search criteria included papers that were published from June 16, 2005 through June 15, 2016) reporting studies conducted in, or review articles and editorials about, the United States. We selected for articles written in English, because this review focuses on publications about the United States. This period spans the majority of the time pregabalin was approved by the US Food and Drug Administration. The selected papers included pregabalin as a core topic as well as a minimum of one formulary restriction policy.

**Table 1:** Search limits & terms*

Category:	Search Limits:
Databases	PubMed
Date range	June 16, 2005–June 15, 2016
Countries	United States
Combinations	Pregabalin *AND* ≥ 1 other relevant term related to restriction policies and health plans)
Topic:	Search Terms:
Drug	Lyrica *OR* pregabalin
Restriction	Restriction
Restriction	Prior authorization
Restriction	Step edit
Restriction	Step therapy
Restriction	Prior approval
Restriction	Quantity limits
Restriction	Fail first requirement
Restriction	Step protocol
Restriction	Cost sharing
Restriction	Cost sharing insurance
Health plan	Health plan
Health plan	Insurance
Health plan	Payer(s)
Health plan	Formulary
Health plan	Benefit design
Other	Access
Other	Health care costs
Other	Cost effectiveness
Other	Health care utilization
Other	Health care expenditures
Other	Cost analysis
Other	Cost utility
Other	Cost containment
Other	Economics
Other	Utilization management

* Boolean operators (to include different spellings and tenses of words) and alternative spellings (e.g. ‘healthcare’ / ‘health care’) were included

The initial literature search was conducted and the abstracts and citations of all identified papers were distributed to all authors. Papers were screened for germaneness to the core topic of describing economic impacts of restriction policies related to pregabalin via discussions between the authors over electronic platforms (e.g. email and webinars) and during meetings convened via teleconferences. For papers of interest, full copies of the articles were distributed amongst the author group and discussed. Eligibility criteria were kept broad to increase the ability to capture all relevant articles. The reference lists cited in each paper were also cross-referenced to identify additional potentially applicable publications. Cross-referenced articles of interest were also provided to the authors for their consideration. Because eight articles were identified by the search, no formal extraction forms were utilized. Since all authors are experienced in the subject matter of this review, no formal training was required.

Two authors conducted an independent quality assessment using the Newcastle-Ottawa assessment scale for cohort articles [24] and the Good Practice Task Force Report recommendations of the International Society for Pharmacoeconomics and Outcomes Research, Academy of Managed Care Pharmacy, and National Pharmaceutical Council [25] to evaluate the quality of the financial model study [26]. Following their individual assessments, both reviewers met, discussed, and agreed upon the ratings. Studies were considered to be of high quality if they were rated six stars or higher. Quality assessment results were shared with all authors. There was consensus agreement by all authors on the selection of publications to be included.

## Results

### Identified articles

**Fig. 1:**
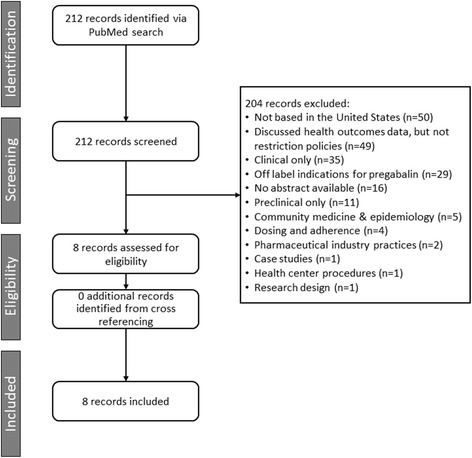
PRISMA diagram of selected publications

Overall, 212 articles were identified by the search and eight of these were determined to fit the inclusion criteria (Fig. 1 and Additional file 1) as well as meet a quality assessment of six or more stars (Additional file 2). Table 2 describes the study designs of articles included in this review. Table 3 summarizes their key healthcare utilization (HCU) and economic cost results.

**Table 2:** Designs of the identified studies evaluating restriction policies for pregabalin

	Margolis et al., 2009 [27]	Margolis, et al., 2010 [28]	Bazalo et al., 2010 [26]	Udall et al., 2014 [30]	Suehs et al., 2014 [31]	Placzek et al., 2014 [29]	Null et al., 2016 [32]	Martin et al., 2016 [33]
Study design (population)	Retrospective database (Medicaid)	Retrospective database (commercial)	Modeling (IMS prescription data)	Retrospective database (commercial)	Retrospective database (Medicare)	Retrospective database (commercial)	Retrospective database (Medicare & commercial)	Retrospective database (commercial)
Number of patients	Restricted, *n* = 424; unrestricted, *n* = 5153	Restricted, *n* = 2084; unrestricted, *n* = 1320	Not applicable	Restricted, *n* = 3876; unrestricted, *n* = 3876	Restricted, *n* = 13,911; unrestricted, *n* = 13,911	Restricted, *n* = 29,283; unrestricted, *n* = 463	Not applicable	Restricted, *n* = 1218; unrestricted, *n* = 1218
Restriction type	PA	PA	PA	ST	ST	PA	ST	Mail order requirement
Conditions evaluated	PHN; pDPN	PHN; pDPN	PHN; pDPN; FM; partial-onset seizures	PHN; pDPN; FM	PHN; pDPN	pDPN; FM	pDPN; FM; PHN	PHN; pDPN; FM; partial-onset seizures

*Abbreviations*: *FM* fibromyalgia, *PA* prior authorization, *pDPN* painful diabetic peripheral neuropathy, *PHN* postherpetic neuralgia, *ST* step therapy

**Table 3:** Differences between restricted and non-restricted cohorts in the identified studies

	Margolis et al., 2009 [27	Margolis, et al., 2010 [28]	Bazalo et al., 2010 [26]	Udall et al., 2014 [30]	Suehs et al., 2014 [31]	Placzek et al., 2014 [29]	Null et al., 2016 [32]	Martin et al., 2016 [33]
Pregabalin utilization	Significantly lower utilization in restricted cohort (*P* < 0.01)	Significantly lower utilization in restricted cohort (*P* < 0.001)	Not applicable (modeling)	Significantly lower utilization in restricted cohort (*P* = 0.008)	Significantly lower utilization in restricted cohort (*P* < 0.001)	No significant difference in utilization in either pDPN (*P* = 0.731) or FM (*P* = 0.192)	Significant increase in commercial plans before (*P* < 0.001), but decrease after (*P* < 0.001) ST started. Small numeric decrease after ST lifted (*P* = 0.568)	Significant decrease with mail order, and increase without (*P* < 0.001)
All-cause pharmacy costs	Not evaluated	Not evaluated	Not evaluated	No significant differences in costs (*P* = 0.197)	No significant differences in costs (*P* = 0.14)	No significant differences in cost for either FM (*P* = 0.835) or pDPN (*P* = 0.846)	Not evaluated	No significant differences in costs (*P* = 0.888)
All-cause healthcare costs	Not evaluated	Not evaluated	Not evaluated	Significantly higher ($1222) in restricted cohort (*P* = 0.016)	No significant difference in costs (*P* = 0.35)	No significant differences in costs for either FM (*P* = 0.667) or pDPN (*P* = 0.625)	Not evaluated	No significant difference (*P* = 0.474)
Disease-related pharmacy costs	Significantly higher ($274) in restricted cohort (*P* < 0.001)	No significant differences in costs (*P* = 0.25)	Potential savings in the restricted plan were < 2%	No significant differences in costs (*P* = 0.120)	Significantly higher ($12) in restricted cohort (*P* < 0.001)	No significant differences in costs for either FM (*P* = 0.948) or pDPN (*P* = 0.946)	Not evaluated	Not evaluated
Disease-related total healthcare costs	Significantly higher ($418) in restricted cohort (*P* < 0.01)	No significant differences in costs (*P* = 0.41)	Not evaluated	Significantly higher ($859) in restricted cohort (*P* = 0.002)	No significant differences in cost (*P* = 0.23)	No significant differences in cost for either FM (*P* = 0.419) or pDPN (*P* = 0.431)	Significantly lower (Medicare, *P* < 0.001) after ST start, but with a rising trend over the analysis period	Not evaluated

*Abbreviations*: *FM* fibromyalgia, *PA* prior authorization, *pDPN* painful diabetic peripheral neuropathy, *PHN* postherpetic neuralgia, *ST* step therapy

### Payer restriction policies for pregabalin: Prior authorization

A few studies have evaluated cost impacts of PA restriction policies for pregabalin. Margolis et al. compiled data from US Medicaid records (2005–2006) of patients with pDPN or PHN and compared states with (2 states, *n* = 424) versus without (4 states; *n* = 5153) PA policies [27]. States with PA policies had a smaller increase in pregabalin use compared with unrestricted states (+9.2% vs +13.6%, respectively, for a difference in difference of −4.4%, *P =* 0.01), with an estimated marginal effect of a 4.0% decrease in the probability of any pregabalin use (*P* = 0.02). However, the likelihood of opioid use in the restricted states (vs. unrestricted states) was significantly higher by 6.5% (*P* < 0.01). In addition, other non-opioid analgesics, “other antidepressants” (i.e. bupropion, citalopram, duloxetine, paroxetine, trazodone, venlafaxine), and anxiolytics had significantly higher probability of being used in PA restricted versus unrestricted states (all *P* < 0.05). In restricted versus unrestricted states, the overall annual per patient pDPN and PHN healthcare costs were $270 higher and after controlling for baseline characteristics, the relative cost increase was estimated to be $418 per patient (both *P* < 0.01). Thus, PA restricted states had relative increases (rather than cost savings) in total PHN and pDPN expenditures (including pharmacy costs) accompanied by greater opioid utilization.

In another study, six health plans with (*n* = 2084) and six plans without (*n* = 1320) PA restriction policies for pregabalin (from 2005 to 2007) were evaluated for potential effects on medication use and healthcare costs for treatment of pDPN and PHN [28]. Consistent with the previous study, pregabalin utilization with PA restriction policies did not increase as much as with non-restricted plans over the post-index period (+7.5% vs +12.8% net unadjusted differences, respectively for a difference in difference of −5.3%, *P* < 0.001). The PA restriction (vs. no restriction) plans had a decreased probability of pregabalin use by 5.0% (adjusted estimated marginal effect *P* < 0.001). Statistically significant relative changes were observed with PA restriction plans in the probability of using other antiepileptic drugs (aside from pregabalin, relative increase of 3.7%) and non-opioid analgesic medications (relative decrease of 5.2%) (both *P* < 0.05). In spite of the relatively lower increase in pregabalin utilization between PA restricted compared with unrestricted plans, no statistically significant cost savings were found in total disease-related healthcare expenditures (*P* = 0.40).

Bazalo et al. simulated scenarios with and without a PA requirement for pregabalin using IMS Health prescription data for patients with FM, pDPN, PHN, and partial onset seizures over a 1-year period [26]. This Excel-based model calculated healthcare and administrative costs for each scenario with PA plans. In the baseline analysis, PA plans had cost differences of 0.4% less than plans without PA requirements (calculated drug acquisition cost of PA $885,564 vs. non-PA $888,822). Sensitivity analyses were conducted with various assumptions (e.g. no PA administration costs, lowering PA approval rate [50% to 10%], raising pregabalin market share without PA [10% to 20%], limiting substitutable therapies to valproate sodium and gabapentin). PA plans using any one of the assumptions ranged from 0.4% to 1.9% less than the cost of no PA plans. In addition, using the assumption of 50% of patients switching to pregabalin over 1 year, a sensitivity analysis found a 0.4% higher cost with PA plans relative to no PA plans. Thus, the investigators concluded that any significant cost savings of PA policies may be offset by administration costs.

Placzek et al. conducted a retrospective, observational analysis of HealthCore Integrated Research Environment claims data (2007–2012) to assess the HCU and economic impact of PA versus no PA policies for pregabalin in patients with FM or pDPN [29]. After propensity score matching, patients were evaluated with (FM *n* = 1852; pDPN *n* = 1040) and without PA requirements (FM *n* = 463; pDPN *n* = 260). From the pre-index date (6 months prior to the index date, defined as the first pharmacy claim for an FM or pDPN medication) to the post-index date (6 months post index date), no significant differences were found between PA and no PA plans in prescription costs (or medical costs (all *P* > 0.05). Moreover, with PA versus non-PA plans, disease-related use of opioids in FM patients was more commonly used (64.6% vs. 57.9%; *P* = 0.0082) and serotonin norepinephrine reuptake inhibitors (SNRIs) in pDPN patients were less commonly used (1.1% vs. 3.1%; *P* = 0.0152).

### Payer restriction policies for pregabalin: Step therapy

To evaluate how ST protocols might affect HCU and costs, Udall et al. conducted a retrospective observational study of Humana claims data (including ST restrictions for pregabalin) and the Thomson Reuters MarketScan Commercial and Medicare Supplemental Database (no ST restrictions) [30]. Patients were adults (aged 18–65 years) with a diagnosis of pDPN, PHN, or FM during 2008 or 2009, with the majority having FM. This study analyzed a 1-year period each before and after the index date (the date of ST protocol implementation). Patients in ST restricted plans and unrestricted plans were matched (*n* = 3876 in each cohort) on diagnosis and geographic region. Before and after ST implementation, a reduction in claims for pregabalin per patient was observed from 3.6 (SD 3.9) to 2.3 (3.6), respectively. For unrestricted plans, claims went from 2.8 (3.1) pre-index to 2.6 (3.3) post-index. Overall, the patients in ST restricted (vs. unrestricted) plans showed significantly greater year-over-year reductions in use (−2.6%; *P* = 0.008) and number of claims (−1.1; *P* < 0.001) for pregabalin from before to after the ST policies were implemented. Using a multiple regression model of medication utilization, the patients in ST restricted (vs. unrestricted) plans were found to have net decreased odds of using pregabalin from 2008 to 2009 (odds ratio [OR] 0.04; 95% confidence interval [CI] 0.02, 0.08; *P* < 0.001), while having increased odds of using gabapentin (OR 2.6; 95% CI 1.72, 3.94), as well as TCAs (OR 2.44; 95% CI 1.48, 4.00), lidocaine or other anesthetics (OR 2.26; 95% CI 1.18, 4.34), SNRIs (OR 1.51; 95% CI 1.06, 2.14), or selective serotonin reuptake inhibitors (SSRIs; OR 2.19; 95% CI 1.42, 3.37) (all *P* < 0.05). When evaluating HCU, significant increases were found with ST restricted (vs. unrestricted) plans in the use of disease-related outpatient visits (difference of +3.6%, *P* = 0.022). When adjusted for demographic and clinical characteristics, the ST restricted (vs. unrestricted) health plans had associated increases in disease-related total healthcare costs (+$859; *P* = 0.002). None of the other costs were significantly different between health plans, suggesting that the pregabalin ST policies are unlikely to yield cost savings.

In another retrospective observational study by Suehs et al., claims data from Humana and Thomson Reuters MarketScan databases were evaluated in older subjects (aged 65 to 89 years) with FM, pDPN, or PHN who were continuously enrolled in a Medicare Advantage Prescription Drug plan from 2008 through 2009 with at least one medical claim or pain intervention within 60 days of diagnosis. The objective of this study was to determine the HCU and expenditures associated with ST requirements for pregabalin compared with unrestricted plans over a 24-month period [31]. From the pre- to post-index periods, the number of members who had a claim for pregabalin numerically decreased (no restrictions 2274 to 2035; ST restriction 1029 to 518) and for gabapentin increased (no restrictions 2828 to 3182; ST restriction 2414 to 3082). Relative to patients in unrestricted plans, patients under ST restrictions had a relative 2.0% decrease in pregabalin utilization and a relative 2.3% increase in the use gabapentin (both *P* ≤ 0.001). A generalized linear mixed model (controlling for age and comorbidities) estimated the OR of patients in ST restricted (vs. unrestricted) plans using pregabalin to be 0.013 (95% CI 0.009, 0.019; *P* < 0.001), while the odds of using gabapentin were higher at 1.908 (95% CI 1.598, 2.276; *P* < 0.001). The odds were also increased in restricted plans for patients using non-opioid analgesics and SSRIs and decreased for other antiepileptic drugs and SNRIs (all *P* < 0.01). With ST restricted (vs. unrestricted) plans (controlling for covariates), annual disease-related pharmacy costs were significantly higher (+$12) (both *P* < 0.001). However, no significant differences were found associated with ST health plans in disease-related healthcare or medical costs (all *P* > 0.05).

In the most recent ST study, Null et al. conducted a retrospective, interrupted time series analysis using data from the Humana Research Database on a ST policy for pregabalin that was implemented and later lifted (in the Medicare plan only) for patients with DPN, FM, and PHN who had a filed a pharmaceutical claim (Medicare Advantage and Pharmacy Benefits, Pharmacy Drug Plan for Medicare, or a commercial plan) [32]. Monthly time series data were evaluated for changes in utilization of pregabalin or of therapeutic alternatives (reported as prescriptions per 100,000 members per month unless otherwise noted), as well as medical and total costs. These data were analyzed during the following time periods: 1) January 2007 to 2009 (prior to the implementation of ST), 2) January 2009 to April 2013 (ST policy in commercial and Medicare plans), 3) May 2013 to April 2014 (after ST policy was no longer in effect in the Medicare plan). Overall, prior to ST implementation, the number of prescriptions increased numerically (+11.9 prescriptions; 95% CI –4.2 to 28.1, *P* = 0.148) in Medicare plans and significantly (+3.5 prescriptions, 95% CI 2.7 to 4.3, *P* < 0.001) in commercial plans. After ST policies went into effect, the trend decreased in the number of pregabalin prescriptions in the Medicare plan (−16.2, 95% CI –37.8 to 5.5, *P =* 0.143), and had an increasing trend after the ST policy was lifted (+8.2 prescriptions, 95% CI –18.4 to 34.7, *P* = 0.546). In the commercial plan, there was a significant decrease in pregabalin prescriptions by −3.6 (95% CI –4.7 to −2.5, *P* < 0.001) after the ST policy was initiated. During the period when the ST policies were lifted in the Medicare but not commercial plans, the commercial plans continued to show a non-significant decrease in pregabalin utilization by −1.3 prescriptions (95% CI –5.9 to 3.2, *P* = 0.568).

In this same study, effects of the ST policy on utilization of other pain drugs were also evaluated [32]. After the ST policy went into effect in Medicare plans, significant changes were found in the utilization per 100,000 members per month of antiepileptic drugs other than pregabalin (gabapentin) (+44.1, 95% CI 27.4 to 60.9), opioids (−52.7, 95% CI –81.5 to −24.0), and SSRIs (−34.0, 95% CI –51.8 to −16.1) (all *P* < 0.001). After ST started in commercial plans, utilization of some of these drugs significantly changed, including opioids (−9.1, 95% CI –14.1 to −4.2), SNRIs (−3.9, 95% CI –6.6 to −1.3), and SSRIs (+10.1, 95% CI 6.8 to 13.5) (all *P* < 0.01). After the ST policy was lifted in Medicare plans, significant decreases occurred in the utilization of antiepileptic gabapentin (−62.6, 95% CI –100.1 to −25.1), opioids (−83.4, 95% CI –161.6 to −5.2), and SSRIs (−62.8, 95% CI –106.1 to −19.6) (all *P* < 0.05). During this same period when ST was lifted in the Medicare plan, the commercial plans (which still had ST policies) had significant decreases in the utilization of SSRI prescriptions (−19.4, 95% CI –31.4 to −7.4, *P* = 0.002).

No statistically significant differences were found in the medical costs after the pregabalin ST policy was implemented in either Medicare or commercial plans or was lifted in the Medicare plan (all *P* > 0.05) [32]. Following ST policy initiation, total healthcare costs (per 1000 members per month) were significantly lower in the Medicare plan (−20,483.2, 95% CI –30,728.6 to −10,237.9, *P* < 0.001), but the decrease was non-significant in the commercial plan (*P* = 0.086). However, the overall trend showed a steady rise in total healthcare cost over the entire analysis period (implementation of ST in both plans and lift of ST in the Medicare plan) with costs in the Medicare plan rising at a steeper slope than the commercial period.

### Payer restriction policies for pregabalin: Mail order requirement

Martin et al. conducted a retrospective analysis (February 1, 2010 through February 28, 2011) of the drug utilization and cost impacts of a pharmacy program that required either switching to mail order of pregabalin to avoid higher member cost sharing or changing to a lower cost alternative medication (brand or generic) that could be filled either at a retail location or via mail order [33]. Patients (≥19 years of age) filed at least one claim for pregabalin for FM, pDPN, PHN, or partial onset seizures during the analysis period. The majority of subjects (77.6%) had FM and were female (76.7%). A logistic regression model was used to propensity score match 1218 patients in each cohort (program cohort had a mail order requirement, non-program did not) based upon demographic and other characteristics (i.e. mail order and pregabalin use, comorbidities, healthcare costs, and HCU prior to the index date [defined as the first pregabalin claim during the identification period]). Prior to the index date, no significant differences were found in the percentage of retail or mail order claims or the use of any alternative medications (all *P* > 0.5). After the program start, the total number of claims for pregabalin (retail and mail order combined) decreased in the program cohort (4.66 pre-index to 3.80 post-index), but increased in the non-program cohort (4.68 to 6.16) (difference in difference *P* < 0.001). In addition, a significantly larger increase was observed in the mail order claims for pregabalin in the program cohort (3.1% pre-index to 48.1% post-index) than in the non-program cohort (2.8% to 9.4%) (difference in difference *P* < 0.001). There was also a greater increase in the percentage of patients who switched to gabapentin in the program cohort (21.1% pre-index to 31.0% post-index) than in the non-program cohort (16.7% to 15.9%) (difference in difference *P* < 0.001), as well as a relative decrease in the use of SSRIs in the program cohort (30.1% to 27.6%) versus non-program cohort (30.0% to 30.7%) (difference in difference *P* = 0.026). Those program members who switched to gabapentin were significant more likely to have high pre-index Charlson Comorbidity Index scores than those who did not switch (1.25 vs. 0.94, *P* = 0.001). Moreover, during the post-index period, patients who switched to gabapentin (vs. those who did not switch) were more likely to have at least one claim for opioids (63.5% vs. 57.3%), TCAs (17.2% vs. 12.1%), or SSRIs (31.8% vs. 25.7%), as well as utilization of at least one healthcare resource, including outpatient visits (83.9% vs. 78.1%), emergency room visits (51.1% vs. 43.5%), and inpatient stays (23.0% vs. 14.8%) (all *P* < 0.05).

The mean total healthcare and medical costs did not significantly increase in either cohort (all *P* > 0.05) [33]. However, the pharmacy costs were significantly higher in both cohorts during the post-index period (pre-index to post-index, respectively: program cohort $7033 to $7853; non-program cohort $7064 to $7854; both *P* < 0.001), but the relative increases were comparable between cohorts (difference in difference *P* = 0.888).

## Discussion

Policies that restrict patient and provider access to specific therapies are best evaluated in the context of overall health, quality of life, and health-related costs. In this review of the published literature on the potential impacts of policies that restrict access to pregabalin, no substantial cost benefits were found in any of the studies identified. The results of the studies identified in this review suggest that PA and ST policies are effective in reducing the utilization of the restricted drug, but in the case of pregabalin, this reduced use does not appear to have a consistent and significant medical cost benefit.

In spite of this, some formularies require PA or a ST edit through one or two other generic drugs before a patient can be reimbursed for pregabalin. Often, this policy includes the use of gabapentin, which is not indicated in the United States for the treatment of pDPN or FM [17–19]. Furthermore, some of the ST studies reviewed also showed increases in the utilization of TCAs, topical anesthetics, SNRIs [30], SSRIs [30, 32], non-opioid analgesics (including some nonsteroidal anti-inflammatory drugs [NSAIDs]) [31], and antiepileptic drugs aside from pregabalin [32], which included some medications without an indication for FM or NeP. Similarly, some of the PA studies found statistically significant increases in the use of non-opioid analgesics, “other antidepressants” (bupropion, citalopram, duloxetine, paroxetine, trazodone, and venlafaxine), and anxiolytics [27], and other antiepileptic drugs (not including pregabalin) [28], including some medications not indicated for NeP and FM. The evidence supporting SSRIs [34], NSAIDs [35], and BZDs [36, 37] for treatment of NeP and FM are inconsistent. In particular, potential for increased use of BZDs could be concerning, given an increasing trend in the number of annual overdose-related deaths with an approximate 5-fold cumulative increase from 2001 through 2014 [38].

Another notable finding observed in two of the studies reviewed is that health plans with, relative to without, PA requirements had lower increases in pregabalin use, but increases in the utilization of opioids in patients with pDPN or PHN [27] and FM [29]. In addition, one study found a decrease in opioid usage in Medicare ST plans, but an increase in opioid utilization in commercial ST plans [32]. In a study of mail order requirements for pregabalin, patients who opted to switch from pregabalin to gabapentin showed a post-index increase in the utilization of opioids [33]. Although more research would be needed to elucidate the potential link between restriction policies against pregabalin and opioid use, observed increases in opioid use should be a consideration when establishing these restriction policies. The 2016 US Centers for Disease Control and Prevention (CDC) opioid prescribing guideline specifically states “… nonopioid pharmacologic therapy [is] preferred for chronic pain” [39]. In addition, the CDC has reported a 200% increase in opioid (including prescription and heroin) overdose deaths since the year 2000, including a 14% increase between 2013 (7.9 deaths per 100,000 persons) and 2014 (9.0 deaths per 100,000 persons) alone [40].

Specifically for chronic NeP, two Cochrane meta-analyses concluded that the literature is inconclusive to support long-term use of opioids in these patients [41, 42]. The Neuropathic Pain Special Interest Group of the IASP published recommendations for the treatment of chronic pain, in which they suggest that the potential long-term safety issues of opioids make them more suitable as second-line therapies for NeP only in patients who have not responded to first-line therapies [6, 7]. Notably, pregabalin was recommended among their first-line therapies, owing to its comparable efficacy and tolerability to gabapentin and more linear pharmacokinetics that make dosing more predictable.

Furthermore, there is little evidence supporting a therapeutic effect of opioids in FM, with many studies finding that opioid use in these patients is no better than standard of care [41, 43], or that opioids may even result in worse outcomes in daily living, function, depression, and insomnia relative to patients taking another therapy [44, 45].

ST policies can reduce health plans’ prescription costs, but little data exist on how these policies may impact quality of care [15, 46]. One challenge of ST policies is they take the medical decision-making out of the hands of the healthcare provider and patient. For example, in a survey of 3929 patients who experienced at least one ST edit for proton pump inhibitors or branded medication, 44% received a therapy other than the one prescribed [47]. Patients who accepted their health plans’ choices of drug reported significantly less satisfaction with the medications than those who received their own first choice (*P* < 0.001). Other patients in this survey sought coverage of their first choice medication (e.g., doctor or pharmacist sought permission [15%]; used another insurance plan [7%]), paid full out-of-pocket cost for their first choice (11%), took no medication at all (11%), or used over-the-counter medication (8%).

The studies reviewed have some limitations. First, the prices and payor coverage of medications in the United States can differ between and even within regions, which can lead to considerable variability in healthcare costs across the country [48]. These regional differences in healthcare costs make it difficult to generalize the cost data from these studies to the entire country. Moreover, the studies reviewed sampled retrospective data from a span of different years. The price of pregabalin has increased over the course of its life cycle. As such, at the times of these studies, the prices of pregabalin were lower than the present day, which can present a challenge for extrapolating the data to the modern medical economic environment. In addition, since most of these studies retrospectively evaluated claims databases, these results are correlational only and a causal effect of these restriction policies on costs or drug utilization cannot be definitively established. Furthermore, these analyses do not evaluate how other policy changes unrelated to pregabalin might have impacted cost and utilization, or how patient or provider decisions based on medical needs or personal preferences may have affected drug selection. For example, prescription benefits (e.g. tier status, copay amounts) can change from year to year, which can potentially change the out-of-pocket costs for brand-name prescription medications. Finally, this search was limited to PubMed, and publications not indexed in that database or cited in the references of identified articles would not have been captured by our search.

## Conclusions

The studies reviewed do not support the use of restriction policies, as a means of cost savings, in regard to pregabalin. Additional studies are needed to evaluate potential relationships between PA policies that restrict pregabalin, and observations in some of the studies reported here of increased opioid prescribing. Taken together, with the fact that restriction policies sometimes include ST edit drugs with off-label indications, it appears that restriction policies for pregabalin should be re-evaluated by payers to determine if they are accomplishing their intended goals.

## Additional files

Additional file 1: Search strategy. (DOCX 12 kb)

Additional file 2: Study quality of included studies based on the Newcastle-Ottawa Scale (DOC 78 kb)

## Abbreviations

CDC: Centers for Disease Control and Prevention
CI: confidence interval
FM: fibromyalgia
HCU: healthcare utilization
IASP: International Association for the Study of Pain
IOM: Institute of Medicine
NeP: neuropathic pain
OR: odds ratio
PA: prior authorization
pDPN: painful diabetic peripheral neuropathy
PHN: postherpetic neuralgia
SNRIs: serotonin norepinephrine reuptake inhibitors
SSRI: selective serotonin reuptake inhibitors
ST: step therapy
TCA: tricyclic antidepressants
