# Supplementary material for: A systematic review of the effectiveness of policies restricting access to pregabalin
Source: BMC Health Serv Res. 2017 Aug 25;17:600. doi: 10.1186/s12913-017-2503-x (PMC6389065; doi:10.1186/s12913-017-2503-x)
Supplement: Supplementary file 1 — Search strategy. (DOCX 12 kb) [file 12913_2017_2503_MOESM1_ESM.docx]

**Additional File 1. Search strategy**

| **Search** | **Query** | **Items Found** |
| --- | --- | --- |
| #1 | (("2005/06/16"[Date - Publication] : "2016/06/15"[Date - Publication]) AND English[Language]) AND (pregabalin OR Lyrica OR pregabalin[MeSH]) | 2113 |
| #2 | (Access OR (“Health care cost*” OR “Healthcare cost*”) OR “Cost effectiveness” OR (“Health care utilization” OR “Healthcare utilization”) OR (“Health care expenditure*” OR “Healthcare expenditure*”) OR “Cost analys*” OR “Cost utility” OR “Cost containment” OR Economic* OR “Utilization management” OR Restriction* OR “Prior authorization*” OR “Step edit*” OR “Step therap*” OR “Step protocol*” OR “Health plan*” OR Insurance OR (Payer* OR Payor*) OR Formular* OR “Benefit design” OR “cost sharing” OR “cost sharing insurance”) | 1276231 |
| #3 | (Access OR “Health care cost” OR “Healthcare cost” OR “Health care costs” OR “Healthcare costs” OR “Cost effectiveness” OR “Health care utilization” OR “Healthcare utilization” OR “Health care expenditures” OR “Health care expenditure” OR “Healthcare expenditures” OR “Healthcare expenditure” OR “Cost analysis” OR “Cost analyses” OR “Cost utility” OR “Cost containment” OR Economics OR Economic OR “Utilization management” OR Restriction OR Restrictions OR “Prior authorization” OR “Step edit” OR “Step edits” OR “Step therapy” OR “Step therapies” OR “Prior approval” OR “Prior approvals” OR “Quantity limit” OR “Quantity limits” OR “Fail first requirement” OR “Fail first requirements” OR “Step protocols” OR “Step protocol” OR “Health plan” OR “Health plans” OR Insurance OR Payer OR Payers OR Payor OR Payors OR Formulary OR Formularies OR “Benefit design” OR “cost sharing” OR “cost sharing insurance”) | 1342496 |
| #4 | #2 OR #3 | 1422414 |
| #5 | #1 AND #4 | 211 |
