# Supplementary material for: A systematic review of the effectiveness of policies restricting access to pregabalin
Source: BMC Health Serv Res. 2017 Aug 25;17:600. doi: 10.1186/s12913-017-2503-x (PMC6389065; doi:10.1186/s12913-017-2503-x)
Supplement: Supplementary file 2 — Study quality of included studies based on the Newcastle-Ottawa Scale (DOC 78 kb) [file 12913_2017_2503_MOESM2_ESM.doc]

# Additional File 1 Study quality of included studies based on the Newcastle-Ottawa Scale

## 4.0 Study quality of cohort studies

| **Author** | **Representativeness of the exposed cohort** | **Selection of the non-exposed cohort** | **Ascertainment of exposure** | **Demonstration that outcome of interest was not present at start of study** | **Comparability of cohorts on the basis of the design or analysis** | **Assessment of outcome** | **Was follow-up long enough for outcomes to occur** | **Adequacy of follow up of cohorts** | **Total score** |
| --- | --- | --- | --- | --- | --- | --- | --- | --- | --- |
| Margolis et al, 2009 | ★ | ★ | ★ | ★ | ★★ | ★ | ★ | ★ | 9 |
| Margolis et al, 2010 | ★ | ★ | ★ | ★ | ★ | ★ | ★ | ★ | 8 |
| Udall et al, 2014 | ★ |  | ★ | ★ | ★★ | ★ | ★ | ★ | 8 |
| Suehs et al, 2014 | ★ |  | ★ | ★ | ★★ | ★ | ★ | ★ | 8 |
| Placzek et al, 2014 | ★ | ★ | ★ | ★ | ★★ | ★ | ★ | ★ | 9 |
| Null et al, 2016 | ★ | ★ | ★ | ★ | N/A | ★ | ★ |  | 6 |
| Martin et al, 2016 | ★ | ★ | ★ | ★ | ★★ | ★ | ★ | ★ | 9 |

*Bazalo et al, 2010: Not applicable as study was a financial model. Reviewers utilized ISPOR AMCP-NPC Good Practice Task Force Report to assess relevance and credibility of model. Model deemed acceptable for study objective.
